# The Effectiveness of Traditional Chinese Medicine Combined With Surgery to Treat Granulomatous Mastitis: A Propensity-Matched Analysis

**DOI:** 10.3389/fonc.2022.833742

**Published:** 2022-02-10

**Authors:** Reziya Sawuer, Chunyu Wu, Zhenping Sun, Sheng Liu

**Affiliations:** ^1^Department of Breast Surgery (Integrated Traditional and Western Medicine), Longhua Hospital, Shanghai University of Traditional Chinese Medicine, Shanghai, China; ^2^Longhua Hospital, Shanghai University of Traditional Chinese Medicine, Shanghai, China

**Keywords:** granulomatous mastitis, surgery, traditional Chinese medicine, clinical outcome, integrated Chinese and Western surgery

## Abstract

**Purpose:**

The etiology and pathology of granulomatous mastitis (GLM) are still unknown. Expert consensus on the treatment of GLM has not been developed. The objective of this study is to study the effectiveness of traditional Chinese medicine (TCM) combined with surgery in treating GLM.

**Materials and Methods:**

A retrospective cohort study was implemented at Longhua Hospital of Shanghai University of Traditional Chinese Medicine in China between September 2019 and August 2021. Female patients were included according to the propensity-score matching (PSM) method and balanced according to age and BMI. Patients with GLM diagnosed by pathology and a course of disease ≥ 6 months were included in this trial. Patients were divided into the TCM alone group or TCM + surgery group.

**Results:**

In total, 168 female patients were assessed and 102 patients were included in the study after PSM (51 in the TCM group and 51 in the TCM + surgery group). The average age of the patients was 32 years (21-47 years). There was no significant baseline characteristics difference between two groups after PSM. The suppuration rate in the TCM + surgery group was less than that in the TCM group (64.7% *vs.* 83.35%, P < 0.05), and the TCM + surgery group had a higher 9-month cure rate than the TCM group (86.3% *vs.* 52.9%, P < 0.05). The full course of disease in the TCM + surgery group was shorter than that in the TCM group (253.9 ± 117.3 days *vs.* 332.5 ± 111.6 days, P < 0.05).

**Conclusions:**

TCM combined with surgery can improve the cure rate and shorten the full course of GLM treatment, indicating surgery should be integrated in the clinical management of GLM.

## Introduction

Granulomatous mastitis (GLM), also known as idiopathic granulomatous mastitis and granulomatous mastitis, is an inflammation in which macrophages and neutrophils infiltrate the breast lobules to form necrotic granulomatous lesions, which was first described by Kessler et al. (1). The typical clinical manifestations of this disease include breast pain, breast mass, nipple depression, nipple overflow, axillary lymph node enlargement, nonlactating breast abscess, fistula, etc. (2, 3). There was no obvious specificity in the clinical manifestations of GLM. Thus, GLM is easily confused with inflammatory breast cancer, and the diagnosis of GLM mainly requires histopathological examination (4–6).

Because the etiology and pathology of GLM are unclear, there is no unified treatment plan to treat GLM. At present, the reported treatment includes surgical treatment and conservative treatments. Although the healing time of surgical treatment is short, the wound complications and recurrence rate are relatively high, which makes surgeons carefully choose this treatment (7, 8). Conservative treatments mainly include antibiotic treatment, steroid treatment, and traditional Chinese medicine (TCM) treatment. The effect of the antibiotic treatment is poor (9). Steroid therapy can cause complications, and serious side effects limit its long-term use (10). TCM treatment, used as a supplementary treatment for evidence-based diseases, has a significant therapeutic effect on inflammatory diseases (11, 12). Additionally, the therapeutic effect of TCM on GLM was recognized (13). A previous study suggested that the 9-month cure rate of TCM was 63%, which was equivalent to that of the TCM surgical treatment (68%) (14). Although the curative effect of TCM was apparent, the treatment time was relatively long. At present, although GLM is a benign lesion, there is no ideal treatment. This disease is difficult to cure, has a high recurrence rate, and causes a tremendous psychological burden to patients (15). Therefore, the present trial intends to study whether TCM combined with surgical treatment can improve the outcome of GLM using the propensity score matching method.

## Materials and Methods

### Patients

A retrospective cohort study was performed at Longhua Hospital of Shanghai University of Traditional Chinese Medicine in China between September 2019 and August 2021. Female patients were included through strict inclusion and exclusion criteria screening.

Inclusion criteria: (I) GLM was diagnosed by pathology (based on the pathological diagnosis of Shanghai Longhua Hospital); (2) Course of disease ≥ 6 months; (3) Non pregnant and nonlactating female patients aged 18-45 years (including 18 and 45 years); (4) There was no severe heart, lung, liver and kidney dysfunction; (5) The patients voluntarily participated in this clinical study, did not participate in other clinical trials and signed informed consent.

Exclusion criteria: (I) patients with serious diseases (such as malignant tumors) or mental diseases that affect their survival; (2) diagnosis of systemic lupus erythematosus, rheumatism, or other known autoimmune diseases; (3) abnormal liver and kidney function (ALT, AST, BUN, etc.) exceeds 20% of the upper limit of normal value and other laboratory indices with clinical significance.

The nonexposure group was treated with oral and external applications of traditional Chinese medicine. The exposure group was treated with TCM combined with surgery. The small incision lesion resection and suture was employed for the surgery operation. Golden ointment was used for external application. The drug use was adjusted appropriately according to the patient’s condition. Oral and external application of traditional Chinese medicine continued until recovery. For the exposure group, oral and external application of TCM was used until no obvious redness, swelling and pain were found in the mass. The patient was then admitted to the hospital for small incision resection and suture. After the operation, the above TCM was taken until the patient recovered.

The formula of traditional Chinese medicine is (addition and subtraction of homemade formula): Radix Bupleuri 6 g, Scutellariae Radix 9 g, Curcumae Radix 9 g, Atractylodes Macrocephala Koidz. 15 g, Poria Cocos (Schw.) Wolf. 15 g, Radix Salviae 15 g, raw Crataegi Folium 15 g, and Herba Taraxaci 30 g. The external application treatment was JinHuang ointment made by our hospital, which is provided by the herbal medicine room of our hospital.

Serum liver and kidney function tests were performed every 3 months during the oral administration of traditional Chinese medicine in the two groups to exclude adverse drug reactions.

### Comparison of Clinical Outcomes Using PSM

The clinical data between the two groups were collected, including general information such as age, height, and weight, marriage and childbearing, time of onset, time interval from the last postpartum to onset, time interval from stopping lactation to onset, course of the disease, lactation, history of previous drug use, history of breast trauma, and history of other diseases. The propensity scores were calculated through logistic regression analysis, including age and BMI. The nearest neighbor matching method to match patients was used through the propensity score matching method. The caliper width was equal to 0.2 times the logit standard deviation of the propensity score. After matching, the statistical significance and standardized differences in the covariate balance were reviewed.

### Evaluation and Follow-Up

The cure rate was calculated at 6 months and 9 months of treatment to evaluate the curative effect. Three and 6 months after the cure, the patient was asked to return to the hospital for an ultrasound review to assess the recurrence rate.

### Standard of Cure

The evaluation criteria for acne mastoid carbuncle (“Diagnosis and Efficacy Criteria for TCM Diseases” version 2017) and the “Consensus of Traditional Chinese Medicine Experts on the Diagnosis and Treatment of Granulomatous Mastitis” issued by the Breast Disease Branch of the Chinese Society of Chinese Medicine in 2017 were used to evaluate the cure criteria. (I) Healed: the mass disappeared, the fistula healed, and the systemic symptoms subsided; ultrasound showed no remaining lesions. (II) Clinical recovery: the systemic symptoms disappeared, the original inflammatory lesions were clinically untouchable, and the ulcers or sores were healed. (III) Improvement: the redness and heat pain disappeared, the mass shrank, and the fistula was nearly healed; ultrasound showed that the heterogeneous hypoechoic area or mixed gyrus area was significantly smaller than before. (IV) Unhealed: the mass did not disappear, the fistula did not heal, and the lesion area was even enlarged; ultrasound showed that the heterogeneous hypoechoic area or mixed back area was identical to the previous observation or enlarged, or the liquid dark area was visible.

The cure (includes healed and clinical recovery mentioned above I and II) rate is the ratio of the number of cured cases in each group to the total number of cases in the group at the sixth and ninth months from the beginning of the enrollment.

The total course of treatment is the first time that B-ultrasound shows no lesions, serum recovery period or postoperative changes since the self-hospital treatment.

**Figure 1:**
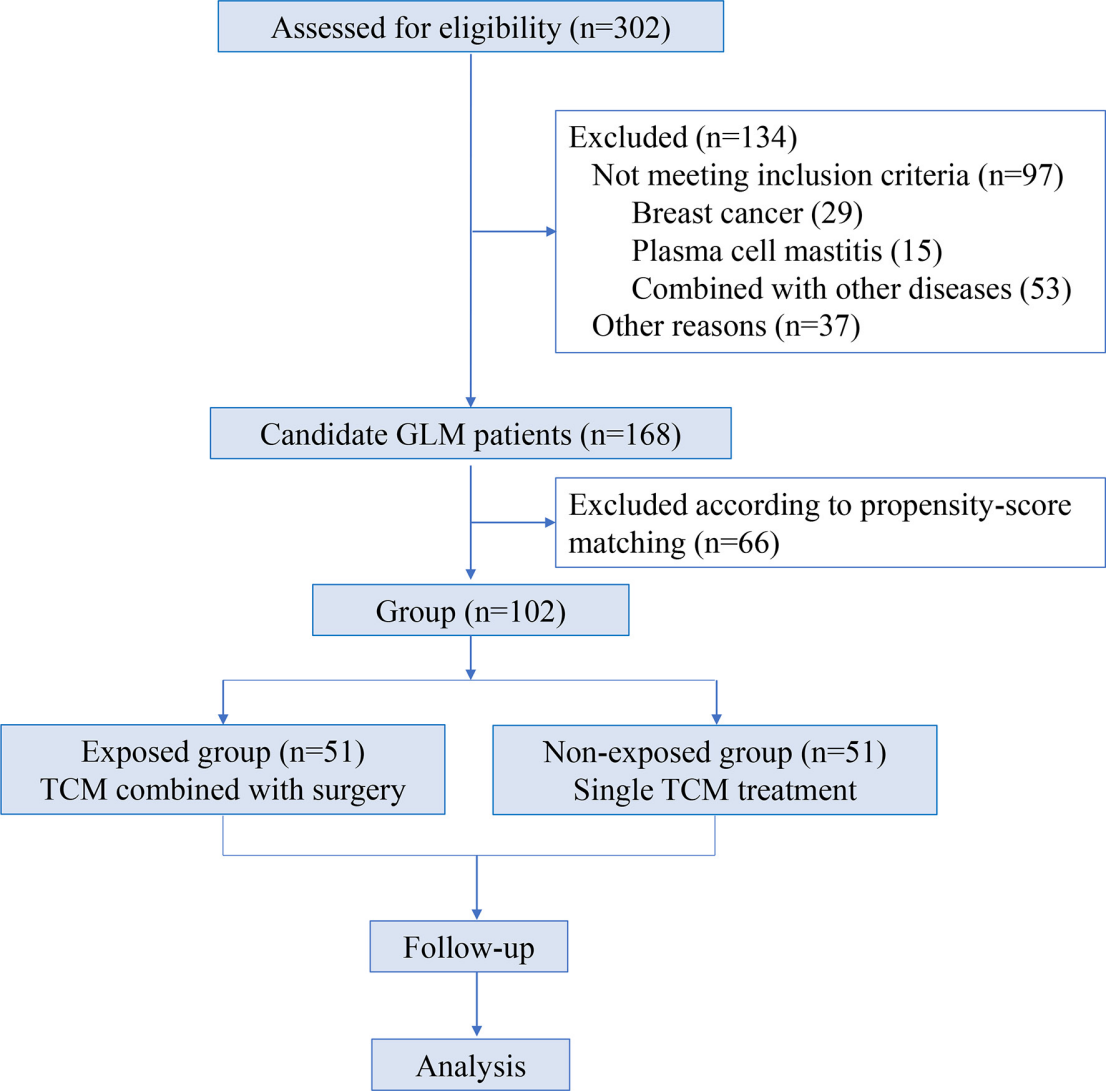
Flow diagram of the trial.

The enrollment and analysis process of GLM patients are shown in **Figure 1**.

### Statistical Methods

A patient follow-up information database was established using the Excel software, and the SPSS 24.0 statistical software was used for statistical data analysis. For the descriptive statistical analysis, the measurement data are expressed as the mean ± standard deviation (x ± s). The count data, i.e., classified data, are represented by the number of cases and the composition ratio. For the comparative analysis of the two groups, the measurement data were measured by t-test or Wilcoxon rank-sum test, and the count data or rank data were compared by chi-square test or rank-sum test. The results were considered statistically significant at P<0.05.

## Results

### Baseline Characteristics Between Two Groups Were Well Balanced Following PSM

In total, 302 female GLM patients were employed at Longhua Hospital of Shanghai University of Traditional Chinese Medicine in China between September 2019 and August 2021. Among them, 168 patients met the inclusion and exclusion criteria. Finally, 102 cases were included and divided into two groups according to the matching propensity score in this study (**Figure 1**).

The clinical data between the two groups before and after matching the propensity score were analyzed, including general conditions such as age, body mass index (BMI), marriage and childbirth, onset time, time interval after last childbirth to onset, time interval between stopping breastfeeding, onset, course of the disease, breastfeeding, history of past drug use, history of breast trauma, and history of other diseases (**Table 1**). Compared with the integrated traditional Chinese and surgery group, the body mass index (BMI) of the patients in the TCM group before the tendency matching was greater (24.9 ± 3.7 *vs.* 24.4 ± 3.5, p=0.019), the proportion of these cases with a history of acute mastitis during lactation was greater (26.5% *vs.* 9.8%, p=0.015), the proportion of these cases with fever symptoms after the onset was higher (25.6% *vs.* 11.8%, p=0.044), and the time from onset to our hospital treatment was shorter (32.1 ± 42.0 *vs.* 62.3 ± 58.7, p=0.002, **Table 1**). After the propensity score matching, the primary conditions of patients between the two groups were balanced, and the difference was not significant.

**Table 1:** Baseline characteristics and propensity score matching.

Types	Full queue			Probability score matching		
	Exposed group (n=51)	Non-Exposed group (n=117)	p	Exposed group (n=51)	Non-Exposed group (n=51)	p
Age*	31.94 ± 4.23	32.06 ± 4.37	0.45	31.94 ± 4.23	33.33 ± 5.41	0.078
Hyperprolactinemia			0.86			0.63
Yes	12 (23.5%)	29 (24.8%)		12 (23.5%)	10 (19.6%)	
No	39 (76.5%)	88 (75.2%)		39 (76.5%)	41 (80.4%)	
History of breast trauma			0.084			0.28
Yes	13 (25.5%)	46 (39.3%)		13 (25.5%)	18 (35.3%)	
No	38 (74.5%)	71 (60.7%)		38 (74.5%)	33 (64.7%)	
History of oral contraceptives			0.52			0.51
Yes	4 (7.8%)	13 (11.1%)		4 (7.8%)	6 (11.8%)	
No	47 (92.2%)	104 (88.9%)		47 (92.2%)	45 (88.2%)	
History of inverted nipples			0.86			0.69
Yes	23 (45.1%)	51 (43.6%)		23 (45.1%)	21 (41.2%)	
No	28 (54.9%)	66 (56.4%)		28 (54.9%)	30 (58.8%)	
History of nipple discharge			0.043			0.135
Yes	13 (25.5%)	15 (12.8%)		13 (25.5%)	7 (13.7%)	
No	38 (74.5%)	102 (87.2%)		38 (74.5%)	44 (86.3%)	
Breastfeeding history						0.22
Yes	43 (84.3%)	104 (88.9%)	0.41	43 (84.3%)	47 (92.2%)	
No	8 (15.7%)	13 (11.1%)		8 (15.7%)	4 (7.8%)	
Acute mastitis			0.044			1.0
Yes	6 (11.76%)	30 (25.6%)		6 (11.76%)	6 (11.76%)	
No	45 (88.24%)	87 (74.4%)		45 (88.24%)	45 (88.24%)	
BMI*	24.35 ± 3.5	24.88 ± 3.7	0.019	24.35 ± 3.5	23.94 ± 2.97	0.53
Postpartum and onset interval (months)	46.63 ± 25.31	43.18 ± 23.62	0.24	46.63 ± 25.31	37.67 ± 28.8	0.15
Interval between weaning and onset (months)	41.58 ± 22.28	34.08 ± 23.28	0.053	41.58 ± 22.28	33.18 ± 22.75	0.19
Accompanying fever after the onset			0.015			0.16
Yes	5 (9.8%)	31 (26.5%)		5 (9.8%)	10 (19.61%)	
No	46 (90.2%)	86 (73.5%)		46 (90.2%)	41 (80.39%)	
Accompanying erythema nodules after the onset			0.63			0.56
Yes	6 (11.76%)	17 (14.5%)		6 (11.76%)	8 (15.69%)	
No	45 (88.24%)	100 (85.5%)		45 (88.24%)	43 (84.31%)	
Lump staging at visit			0.23			0.12
Lump stage	31 (60.78%)	51 (43.6%)		31 (60.78%)	22 (43.14%)	
Abscess	10 (19.61%)	34 (29.1%)		10 (19.61%)	8 (15.69%)	
Rupture period	7 (13.73%)	21 (17.9%)		7 (13.73%)	15 (29.41%)	
Post-collapse	3 (5.88%)	11 (9.4%)		3 (5.88%)	6 (11.76%)	
Interval between onset and treatment in our hospital (days)	62.31 ± 58.69	32.14 ± 42.02	0.002	62.31 ± 58.69	64.98 ± 40.76	0.4

*The matching factor.

### TCM Combined With Surgery Reduce the Suppuration Rate

**Figure 2:**
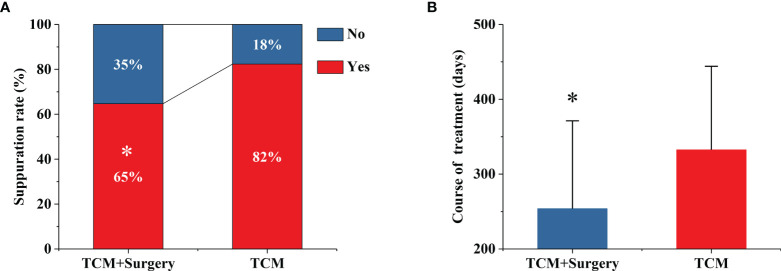
TCM combined with surgery reduce the suppuration rate **(A)** and total duration of treatment **(B)**. *P < 0.05 *vs.* TCM group.

During the treatment period, there were 33 cases and 42 cases of purulent TCM combined with surgery and TCM. The proportion of purulent patients in the TCM-surgery group was significantly less than that in the TCM group (64.7% *vs.* 83.4%, P=0.043) as shown in **Figure 2A**.

### TCM Plus Surgery Significantly Reduced Total Duration of Treatment

The total treatment durations of the exposed group and the nonexposed group were 254 ± 117 days and 333 ± 112 days, respectively (**Figure 2B**). The median days of the treatments between TCM plus surgery and TCM were 260 and 330 days. Compared with the TCM group, the TCM-surgery group had a significantly shorter total course time with a p value 0.001 (**Figure 2B**).

### TCM Combined With Surgery Increased Cure Rate

**Figure 3:**
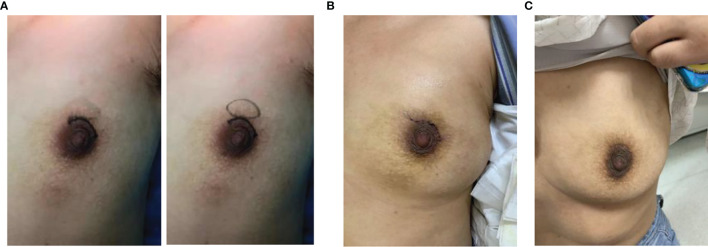
Typical representative photos of GLM before surgery **(A)**, one week after surgery **(B)**, and one month after surgery **(C)**.

According to the evaluation criteria for acne mastoid carbuncle approved by the Breast Disease Branch of the Chinese Society of Chinese Medicine in 2017, only 9 (17.6%) patients in the non-exposed group were healed after 9 months of treatment. Interesting, in the exposed group, one (2.0%) patient was healed within 3 months of treatment, and 14 (27.5%) patients were healed at 9 months. In addition, the clinical recovery cases of the non-exposure group at 3, 6 and 9 months of treatment were 1 (2.0%), 7 (13.7%) and 18 (35.3%), respectively. While, the clinical recovery cases of the exposure group at 3, 6 and 9 months of treatment were 14 (27.5%), 20 (39.2%) and 30 (58.8%), which were significantly higher than the non-exposed group (P<0.001). In summary, the number of cured cases at 3 months, 6 months and 9 months of TCM combined with surgery was 15 cases (29.4%), 26 cases (50.1%), and 44 cases (86.3%), respectively. Correspondingly, there were 1 (1.96%), 7 (13.7%), and 27 (52.9%) cured cases in the TCM group at 3 months, 6 months and 9 months, respectively (**Table 2**). During the three different follow-up times, the TCM-surgery group had a significantly higher cure rate than the TCM group (P<0.01, **Table 2** and **Figure 3**).

**Table 2:** Cure cases (rate) between the two groups.

Groups	Types	3 months	6 months	9 months
Exposed group	Healed	1 (2.0%)	6 (11.8%)*	14 (27.5%)
	Clinical Recovery	14 (27.5%)***	20 (39.2%)**	30 (58.8%)*
	Improvement	34 (66.7%)***	25 (49.0%)***	6 (11.8%)***
	Unhealed	2 (3.9%)	0 (0%)	1 (2.0%)
	Total cure	15 (29.4%)***	26 (50.1%)***	44 (86.3%)***
Non-Exposed group	Healed	0 (0%)	0 (0%)	9 (17.6%)
	Clinical Recovery	1 (2.0%)	7 (13.7%)	18 (35.3%)
	Improvement	48 (94.1%)	42 (82.4%)	24 (47.1%)
	Unhealed	2 (3.9%)	2 (3.9%)	0 (0%)
	Total cure	1 (2.0%)	7 (13.7%)	27 (52.9%)

*P < 0.05, **P < 0.01, ***P < 0.001 vs. Non-Exposed group.

### No Significant Adverse Reactions Between the Two Groups

During the treatment period in the exposure group, 3 patients developed wound fluid accumulation, no obvious adverse reactions were found in the non-exposed group. However, there was no significant difference in adverse reactions between the two groups during treatment (P=0.24).

## Discussion

GLM, which is a chronic inflammatory benign disease, has a higher incidence among Han Chinese women, and its incidence accounts for approximately 3.5% of benign breast diseases in women (16). GLM tends to occur in women of childbearing age, and most patients have a history of pregnancy and breastfeeding (17). In this study, all patients were married women of childbearing age, had an average age of 32 years (21-47 years), and had a history of pregnancy. Moreover, approximately 88% of the patients had a history of breastfeeding. These results are consistent with previous reports. In addition, the average time between the onset of the patient and the last postpartum period was 42 months, and 11.8% of the patients had a history of acute mastitis.

The etiology and pathogenesis of granulomatous lobular mastitis is not yet fully understood. Some researchers believe that the pathogenesis of GLM is due to ductal epithelial damage, which transfers luminal secretions to lobular connective tissue, causing local inflammation of the connective tissue, which in turn allows macrophages and lymphocytes to migrate to this area, causing local granulomatous inflammation (18). The factors that ultimately induce ductal epithelial injury include pregnancy, breastfeeding, history of trauma, hyperprolactinemia, and obesity (18). In this study, 21.6% of patients had high prolactin, 30.1% of patients had breast trauma before the onset, 8.3% of patients had oral contraceptives, 43.1% of patients had inverted nipples, and the average BMI index of these cases was ≥23.9. The results of this study consistent with that of previous studies, suggest that pregnancy, breastfeeding, trauma history, obesity and other factors are risk factors for GLM (18).

There is no clear consensus on the treatment of GLM. At present, the most reported treatments are drug treatment and surgical treatment. Grover et al. believed that steroids or immunomodulatory drugs are used as conventional treatments, and surgical resection is widely adopted as the last treatment for refractory cases (19). Zhou et al. also suggested that drug treatment was recommended as a priority (20). If the patient has a relapse or the drug is not effective, surgery will be used for treatment. However, other researchers believe that the therapeutic effect of immunomodulatory (steroid) therapy is usually poor (21). Therefore, surgical treatment is still one of the main ways to treat GLM abroad. For surgical treatment, Zhang et al. believed that appropriate acute inflammation control methods should be used before surgery, and appropriate surgical methods should be selected after the inflammation has been controlled (13). This strategy has a better therapeutic effect than the traditional extensive resection; i.e., its cosmetic effect is good, the recovery time is short, and the prognosis is better. Wang et al. found that surgery after steroid treatment has a faster curative effect and a lower recurrence rate than steroid treatment alone (22). Zhang et al. found that TCM YangHe decoction combined with surgical treatment had a higher cure rate and a lower recurrence rate than surgical treatment alone (23). Zuo et al. also believed that the treatment of GLM with integrated traditional Chinese and Western medicine could improve the aesthetics of both breasts, thus it is worthy of clinical recommendation (24). A recent review reveals that the effectiveness of TCM plus surgery in the treatment of GLM is similar to that of surgical operation, but TCM plus surgery can significantly improve the satisfaction of patients with breast shape (25). Therefore, the consensus of experts on diagnosis and treatment of granulomatous lobular mastitis in traditional Chinese Medicine was initiated by the China Association of Chinese Medicine (CACM) (26). Although Chinese medicine has a clear curative effect, the treatment time of traditional Chinese medicine is long, and most patients suffer from long-term dressing changes. Therefore, traditional Chinese medicine combined with surgery was used to treat GLM. In the early stage of the disease, TCM was used to reduce the mass and limit the inflammation, and finally the appropriate operation was performed to reduce the damage to the breast shape. Finally, the purpose of shortening the course of treatment and reducing the pain caused by dressing changes was achieved.

Traditional Chinese medicine doctors believes that granulomatous lobular mastitis belongs to the category of “acne breast carbuncle”. There is no clear name of GLM in ancient books. According to its special clinical manifestations and pathogenesis, Professors Gu Bohua and Lu Deming of Shanghai School of Gu’s Surgery proposed the name “acne breast carbuncle” in 1980 (27). They believe that GLM first has nipple deformity or duct dilation and subsequently stagnation of liver qi, failure to follow blood, stagnation of qi and blood stasis, agglomeration into masses, long-term depression, and heat stagnation because of emotional discomfort, which result in meat rot and abscess. Eventually, it becomes a fistula after ulceration. In this study, TCM combined with surgery was used to treat GLM, and Chinese medicines such as Radix Bupleuri, Curcumae Radix, Radix Salviae, and Herba Taraxaci were given to soothe the liver, clear heat, promote blood circulation and remove blood stasis. The local lesions were excised and sutured approximately 4.7 months after the oral administration of TCM. The results of the study suggest that the TCM-surgery group had a significantly lower purulent lesion rate than the TCM group. This result indicates that the use of surgical treatment at the appropriate time can significantly reduce local suppuration, which shortens the course of treatment and reduces pain. The cure rates at 3 months, 6 months and 9 months in the TCM-surgery group were significantly better than those in the TCM group. Moreover, the total treatment course of TCM with surgery was significantly shorter than that of TCM. During the treatment period, there were 3 patients with wound effusion in the traditional Chinese medicine combined with the operation group after surgery. After symptomatic extraction of the effusion and Chinese medicine treatment, the wound healed after an average delay of 2 weeks. There were no obvious adverse reactions in the traditional Chinese medicine group. Therefore, Chinese medicine combined with surgery to treat GLM is worth recommending in clinical practice.

The present study has some limitations. First, this investigation was a retrospective cohort study, and there may be research bias. Second, the sample size in this study was relatively small, and the conclusions of this study must be verified by further multi-center clinical trials with larger sample sizes. Third, the follow-up time of this study was not sufficiently long, and longer clinical follow-up is required for these patients.

## Conclusions

In summary, Chinese medicine combined with surgical treatment can increase the cure rate of GLM, shorten the course of treatment, and reduce the rate of suppuration of local lesions. Therefore, this treatment strategy is worthy of clinical recommendation and is expected to become a comprehensive treatment for GLM.

## Data Availability Statement

The original contributions presented in the study are included in the article/supplementary material. Further inquiries can be directed to the corresponding authors.

## Ethics Statement

The studies involving human participants were reviewed and approved by the clinical trial ethics committee in Longhua Hospital of Shanghai University of Traditional Chinese Medicine. Written informed consent for participation was not required for this study in accordance with the national legislation and the institutional requirements.

## Author Contributions

SL and ZS designed the research. CW and RS performed the research. CW and RS contributed analytic tools. CW and RS wrote the paper. SL and ZS reviewed the paper. All authors contributed to the article and approved the submitted version.

## Funding

This study was supported by grants from the Shanghai Municipal Health Commission (Grant Number ZY3-LCPT-2-1002), and Shanghai Municipal Science and Technology Commission (Grant Number 20Z21900300), as well as Shanghai Hospital Development Center (No. SHDC2020CR1050B).

## Conflict of Interest

The authors declare that the research was conducted in the absence of any commercial or financial relationships that could be construed as a potential conflict of interest.

## Publisher’s Note

All claims expressed in this article are solely those of the authors and do not necessarily represent those of their affiliated organizations, or those of the publisher, the editors and the reviewers. Any product that may be evaluated in this article, or claim that may be made by its manufacturer, is not guaranteed or endorsed by the publisher.

## Abbreviations

GLM, granulomatous mastitis; TCM, traditional Chinese medicine.
